# Untargeted serum metabolomics and air pollution in Parkinson’s disease

**DOI:** 10.1038/s41531-026-01451-3

**Published:** 2026-07-01

**Authors:** Dayoon Kwon, Kimberly C. Paul, Yuyuan Lin, Danielle N. Thordarson, Jun Wu, Jason Su, Dean P. Jones, Jeff M. Bronstein, Beate Ritz

**Affiliations:** 1 Department of Epidemiology and Population Health, School of Medicine, Stanford University, Stanford, CA USA; 2 Department of Neurology, David Geffen School of Medicine, University of California, Los Angeles, CA USA; 3 Department of Epidemiology, Fielding School of Public Health, University of California, Los Angeles, CA USA; 4 Department of Environmental and Occupational Health, School of Population and Public Health, University of California, Irvine, CA USA; 5 Department of Environmental Health Sciences, University of California, Berkeley, CA USA; 6 Department of Medicine, School of Medicine, Emory University, Atlanta, GA USA

**Keywords:** Biomarkers, Diseases, Environmental sciences, Risk factors

## Abstract

Mechanisms linking air pollution to Parkinson’s disease (PD) remain unclear despite consistent toxicological and epidemiological evidence. In this exploratory study, we investigated metabolic disturbances associated with air pollution in PD using untargeted metabolomics. Serum samples were collected from 616 PD patients and 271 controls in central California, a region with high air pollution. Exposures to particulate matter (PM_2.5_), carbon monoxide, and nitrogen dioxide were estimated for the 1-, 3-, and 5-year periods prior to serum collection using three modeling approaches: chemical transport, dispersion, and land use regression. We performed a metabolome-wide association study using liquid chromatography-high-resolution mass spectrometry (2716 HILIC and 2046 C18 features), followed by pathway enrichment analysis. We identified 101 annotated metabolites and 23 metabolic pathways associated with air pollution exposure, particularly PM_2.5_ and traffic-related pollutants. Control profiles aligned with prior studies, and PD patients showed similar disruptions. Air pollution was associated with inflammation-related lipid metabolism (reduced arachidonate; elevated leukotriene), membrane phospholipid remodeling (reduced choline phosphate and LysoPC), amino acid metabolism (reduced aspartate and serine), and dopamine biosynthesis (reduced tyrosine) in PD patients. These findings suggest that air pollution may contribute to PD through inflammation, oxidative stress, and mitochondrial dysfunction.

## Introduction

Epidemiological evidence increasingly associates air pollution with Parkinson’s disease (PD), particularly long-term exposure to particulate matter (PM_2.5_), carbon monoxide (CO), and nitrogen dioxide (NO_2_), key markers indicative of traffic-related air pollution (TRAP)^1,2^. However, the biological pathways underlying these relationships remain largely unknown. Untargeted metabolomics, a powerful analytical technique capable of simultaneously profiling thousands of metabolites, has emerged as an important method for identifying exposure biomarkers and elucidating biological pathways affected by environmental exposures. Recent air pollution metabolomics studies consistently highlight perturbations in metabolic pathways linked to inflammation, oxidative stress, and energy metabolism^3,4^. Similarly, PD metabolomics research has identified disruptions in lipid metabolism and amino acid pathways that are critical to brain health and mitochondrial function^5^.

Mechanistic studies in animal models and post-mortem human tissue indicate that PM and traffic-derived pollutants can reach the brain via systemic circulation, the olfactory tract^6^, and disruption of the blood-brain barrier^7^, where they trigger microglial activation, neuroinflammation, oxidative damage, and mitochondrial impairment in dopaminergic neurons of the substantia nigra^8–10^. Untargeted metabolomics provides a unique window into systemic perturbations that may contribute to these processes, enabling identification of exposure-responsive biological pathways that may distinguish disease-relevant from non-pathological responses.

Building upon these prior findings, we conducted a metabolome-wide association study (MWAS) within a case-control study located in central California, an area known for high levels of air pollution. This study aimed to (1) identify serum-measured metabolic disturbances associated with PM_2.5_ and TRAP exposure (measured by CO and NO_2_) among PD patients and controls; (2) compare findings with existing air pollution metabolomics studies; and (3) provide biological insights into mechanisms linking air pollution exposure to PD.

## Results

### Participant characteristics

The mean age of PD patients in the Parkinson’s Environment and Gene (PEG) study was 67 years at diagnosis and 70 years at time of serum sample collection, with an average PD duration of 3 years (Table 1). Most patients were men (63%), non-Hispanic White (76%), and never-smokers (53%). Controls had a mean age of 66 years at the time of interview and serum collection. The control group was similarly composed of men (62%) and had similar education levels, but had a higher proportion of non-Hispanic White individuals (86%) and a greater percentage of current or former smokers (52%). Most controls with available biosamples participated in the PEG1 wave.

**Table 1:** Characteristics of PEG study participants with metabolomics data

	No. (%) of participants	
**Characteristic**	**Controls (***n* **=** **271)**	**PD patients (***n* **=** **616)**
Age at diagnosis/interview, mean (SD), *y*	66.0 (11.5)	66.9 (10.4)
Age at sample collection, mean (SD), *y*	66.4 (13.1)	69.9 (10.0)
PD duration, mean (SD), *y*	-	3.0 (2.6)
Gender		
Male	169 (62.4%)	390 (63.3%)
Female	102 (37.6%)	226 (36.7%)
Race and ethnicity		
White	234 (86.3%)	468 (76.0%)
Latino	2 (0.7%)	105 (17.0%)
Native American	23 (8.5%)	25 (4.1%)
Asian	3 (1.1%)	13 (2.1%)
African American	9 (3.3%)	5 (0.8%)
Education, mean (SD), *y*	14.5 (3.1)	13.8 (4.5)
Smoking status		
Never	129 (47.6%)	327 (53.1%)
Former	126 (46.5%)	267 (43.3%)
Current	16 (5.9%)	22 (3.6%)
Study wave		
PEG1	186 (68.6%)	275 (44.6%)
PEG2	85 (31.4%)	341 (55.4%)
Year of sample collection, mean (SD), *y*	2008.0 (6.5)	2008.8 (5.0)

### Air pollutant distributions and correlations

The mean estimated air pollutant exposure levels in the year prior to serum sample collection were 12.7 μg/m^3^ for PM_2.5_ (chemical transport model, CTM) and 3.7 ppb for CO (California Line Source Dispersion Model, version 4, CALINE4) (Supplementary Table 2). The exposure distributions were similar for longer averaging periods (e.g., 3 and 5 years prior). CO (CALINE4) was moderately correlated with PM_2.5_ (CTM) (*ρ* = 0.59–0.64) and with NO_2_ (land use regression, LUR) (*ρ* = 0.62–0.64). These moderate correlations reflect differences in source coverage: CALINE4-based CO captures only local vehicle exhaust within 1.5 km of the residence, whereas PM_2.5_ (CTM) and NO_2_ (LUR) represent total concentrations from all sources, though NO_2_ more strongly reflects traffic-related air pollution (Supplementary Fig 2). PM_2.5_ (CTM) and PM_2.5_ (LUR) were also only moderately correlated (*ρ* = 0.49–0.57), highlighting differences between the CTM and CALINE4 models used for primary analyses and the LUR-based models used only for comparison.

### Metabolome-wide association study (MWAS)

**Fig. 1:**
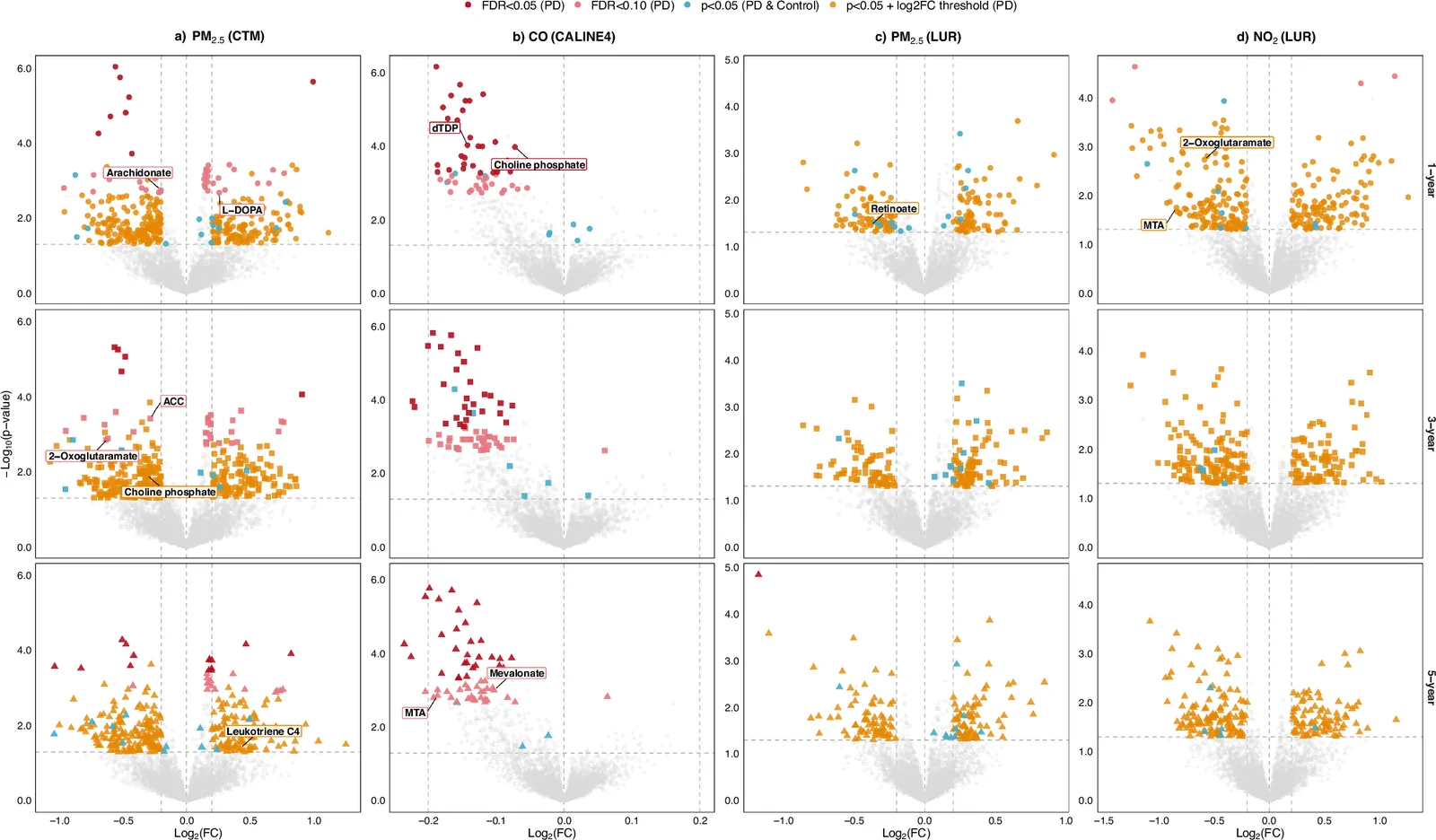
Metabolomic features associated with air pollution exposure in Parkinson’s disease patients. Volcano plots showing log_2_ fold change (log_2_FC, x-axis) versus -log_10_ *p*-value (y-axis) for each metabolite feature in metabolome-wide associations between serum metabolites and air pollution exposure in PD patients. Panels show four exposure models: **a** PM_2.5_ estimated by a chemical transport model (CTM); **b** carbon monoxide (CO) by the California Line Source Dispersion Model, version 4 (CALINE4); **c** PM_2.5_ by land use regression (LUR); and **d** nitrogen dioxide (NO_2_) by LUR. Within each panel, the three rows correspond to 1-year, 3-year, 5-year average exposures prior to serum collection. Point shapes indicate the exposure window (circles, 1-year; squares, 3-year; triangles, 5-year). Point colors indicate statistical significance: red, false discovery rate (FDR) < 0.05 in PD patients; pink, FDR < 0.10 in PD patients; yellow, nominal *p* < 0.05 in PD patients with |log_2_FC | >0.2; blue, nominal *p* < 0.05 in both PD patients and controls. Annotated metabolite features are labeled within each panel. ACC 1-Aminocyclopropanecarboxylate, MTA 5’-Methylthioadenosine, PD Parkinson’s disease, PM_2.5_ particulate matter ≤2.5 µm.

We first examined air pollution-metabolite associations only in PD patients using untargeted MWAS. For PM_2.5_ (CTM), numerous features were identified in C18 mode (FDR < 0.05: 5–14; FDR < 0.10: 29–43; *p* < 0.05 with replication: 221–255) and in HILIC (*p* < 0.05 with replication: 138–162) across 1-, 3-, 5-year exposure averages (Supplementary Table 3). None of the FDR < 0.05 features were annotatable; four metabolites passed FDR < 0.10, including arachidonate and 3,4-dihydroxyphenylalanine (L-DOPA). Arachidonate was negatively associated with 1-year exposure (log_2_FC = −0.20, 95% CI = −0.33, −0.08; Fig. 1) and remained negative at 3- and 5-year exposures at the nominal threshold (*p* < 0.05 with replication). Among additional annotated metabolites meeting the nominal threshold, leukotriene C4 was positively associated with 5-year exposure (log_2_FC = 0.42, 95% CI = 0.02, 0.83); aspartate (e.g., 3-year: log_2_FC = −0.29, 95% CI = −0.43, −0.14), serine (3-year: log_2_FC = −0.10, 95% CI = −0.19,−0.02), and choline phosphate (e.g., 3-year: log_2_FC = −0.31, 95% CI = −0.55, −0.07) were negatively associated, particularly with longer-term exposures (Supplementary Data 1).

For CO (CALINE4) in PD patients, we identified a larger number of features in HILIC (FDR < 0.05: 29–31; FDR < 0.10: 55–66; *p* < 0.05 with replication: 167–190) and C18 (*p* < 0.05 with replication: 42–46) across the three exposure windows (1-, 3-, and 5-year). Two annotated metabolites passed FDR < 0.05 across all three exposure averages: dTDP (e.g., 1-year: log_2_FC = −0.14, 95% CI = −0.21, −0.07) and choline phosphate (e.g., 1-year: log_2_FC = –0.07, 95% CI = −0.11, −0.04), both consistently showing lower abundance with higher CO exposure.

Using alternative exposure models in PD patients, we found only one metabolite feature that met FDR < 0.05 for PM_2.5_ (LUR) in HILIC and four metabolite features at FDR < 0.10 for NO_2_ (LUR) in HILIC. Several nominal associations were consistent in direction with those observed using the CTM model: for PM_2.5_ (LUR), arachidonate was negatively associated with exposure (1-year: log_2_FC = −0.12, 95% CI = −0.22, −0.02). Retinoate (1-year: log_2_FC = −0.38, 95% CI = −0.74, −0.03) and LysoPC(16:0) (1-year: log_2_FC = −0.07, 95% CI = −0.14, −0.001) were also negatively associated with exposure. For NO_2_ (LUR), tyrosine (1-year: log_2_FC = −0.17, 95% CI = −0.31, −0.02) and methionine (3-year: log2FC = −0.20, 95% CI = −0.34, −0.06) were negatively associated with this exposure. In sensitivity analyses, additionally adjusting for levodopa equivalent daily dose (LED), associations for all annotated metabolites remained unchanged (Supplementary Table 4).

In controls, only one metabolite in the C18 mode met the FDR < 0.05 threshold (for CO [CALINE4] across all exposure windows and PM_2.5_ [LUR] at 5-years), and a small number of additional features met FDR < 0.10 (1–5 in C18 and 1–2 in HILIC across pollutants and exposure windows; Supplementary Table 5); none of these features could be annotated. As a result, cross-group comparisons were limited to features with nominal statistical significance (*p* < 0.05 with replication; Supplementary Table 6). Effect estimates were moderately to strongly correlated for PD patients and controls, particularly with the PM_2.5_ (LUR), suggesting overlap in metabolic responses to particulate exposure (Supplementary Fig 3). Two annotated metabolites were reduced in both PD patients and controls with PM_2.5_ (LUR) exposure: arachidonate (controls, 3-year: log_2_FC = −0.17, 95% CI = −0.30, −0.03) and choline phosphate (controls, 3-year: log_2_FC = −0.24, 95% CI = −0.41, −0.08).

Several annotated metabolites in controls showed consistent associations across exposure windows: linoleate (1-year: log_2_FC = −0.22, 95% CI = −0.42, −0.03) and gamma-linolenate (3-year: log_2_FC = −0.27, 95% CI = −0.48, −0.06) decreased with PM_2.5_ (LUR) (Supplementary Fig 4).

### Pathway enrichment analysis

**Fig. 2:**
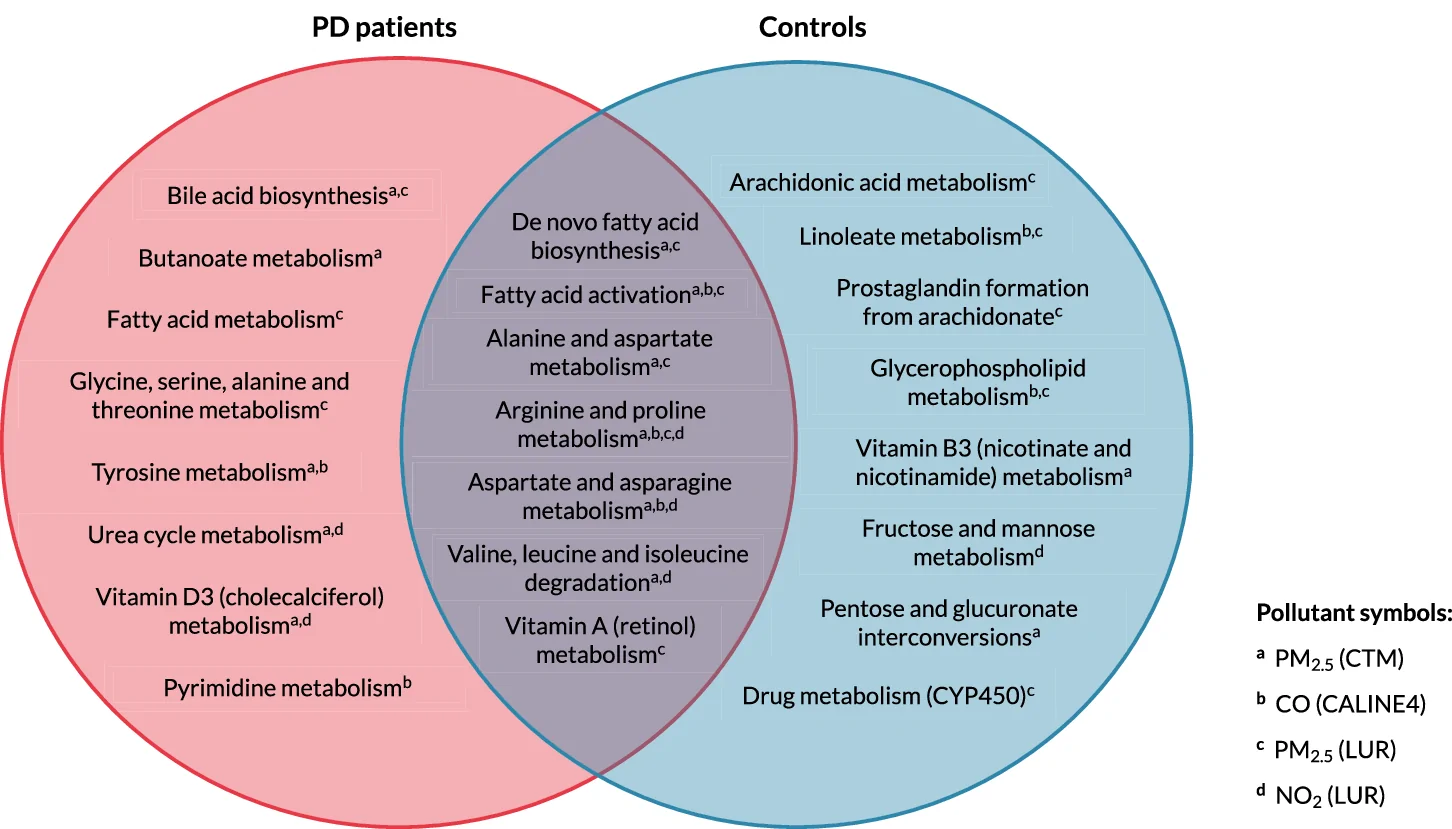
Shared and group-specific metabolic pathways enriched by air pollution exposures in PD patients and controls. Venn diagram comparing metabolic pathways significantly enriched among serum metabolites associated with air pollution exposure in PD patients (left, red) and controls (right, blue), with shared pathways shown in the central overlap. Pathways were included if they contained ≥5 metabolites associated with air pollution and showed pathway overrepresentation at *p* < 0.05. Each pathway name is annotated with one or more superscript letters indicating the air pollution exposure model(s) responsible for its enrichment within that group: **a** PM_2.5_ estimated by a chemical transport model (CTM); **b** carbon monoxide (CO) by the California Line Source Dispersion Model, version 4 (CALINE4); **c** PM_2.5_ by land use regression (LUR); and **d** nitrogen dioxide (NO_2_) by LUR. PD Parkinson’s disease, PM_2.5_ particulate matter ≤2.5 µm.

Of the 23 unique metabolic pathways enriched (≥5 air pollution-associated metabolites; *p* < 0.05), 7 were shared between PD patients and controls, 8 were enriched only in PD patients and 8 only in controls (Fig. 2). Shared pathways spanned lipid biosynthesis (e.g., de novo fatty acid biosynthesis) and amino acid metabolism (e.g., alanine and aspartate metabolism), and vitamin A (retinol) metabolism, driven primarily by PM_2.5_ (LUR) (Supplementary Fig 5).

Pathways enriched only in PD patients extended these themes with additional broader lipid metabolism (e.g., fatty acid metabolism), additional amino acid catabolism (e.g., glycine, serine, alanine and threonine metabolism) and dopaminergic signaling (e.g., tyrosine metabolism) disturbances with exposure. Pathways we found enriched in controls only showed a profile centered on inflammation- and phospholipid-related lipid metabolism (arachidonic acid metabolism, linoleate metabolism, prostaglandin formation from arachidonate, and glycerophospholipid metabolism).

## Discussion

**Fig. 3:**
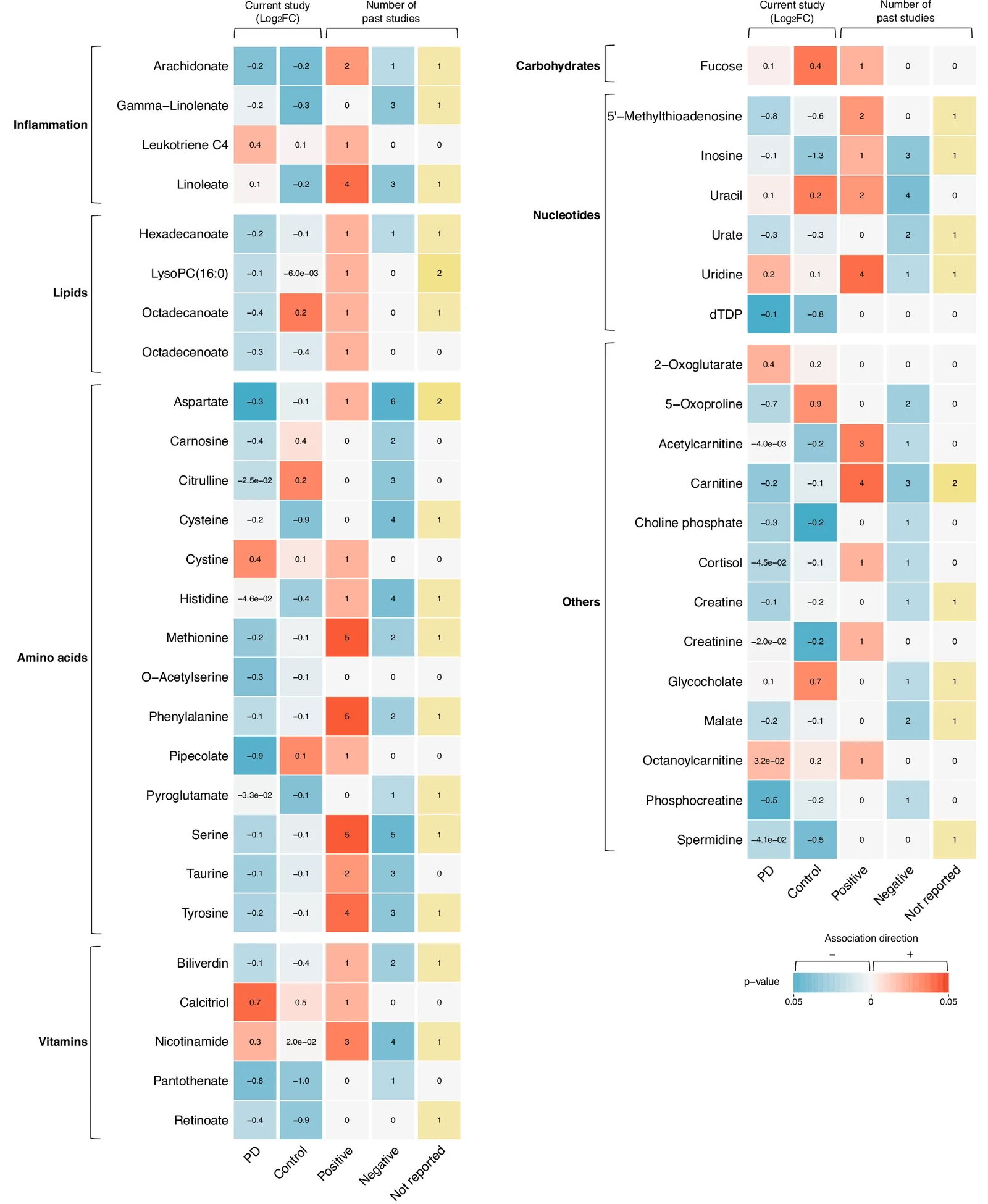
Annotated metabolites associated with air pollution exposure in Parkinson’s disease, compared with prior serum and plasma metabolomics studies. Heatmap summarizing serum metabolite associations with air pollution exposure (PM_2.5_, carbon monoxide [CO], or nitrogen dioxide [NO_2_]) in PD patients and controls from the current study, alongside counts of prior studies reporting the same associations. Metabolites are grouped by biological category (inflammation, lipids, amino acids, vitamins, carbohydrates, nucleotides, and others). For the current study, the two “log_2_FC”columns (PD and Control) show effect estimates: red, positive association (metabolite increased with higher air pollution exposure); blue, negative association (metabolite decreased with higher air pollution exposure); darker shades indicate stronger statistical significance (lower p-value); numeric values within cells are the log_2_ fold change (log_2_FC). The three “Number of past studies” columns summarize associations reported across 29 previous serum or plasma metabolomics studies of air pollution: “Positive” and “Negative” indicate the reported direction of association, and “Not reported” indicates studies that reported an association without specifying direction; color intensity corresponds to the number of studies. dTDP thymidine 5’-diphosphate, LysoPC lysophosphatidylcholines, PD Parkinson’s disease, PM_2.5_ particulate matter ≤2.5 µm.

**Fig. 4:**
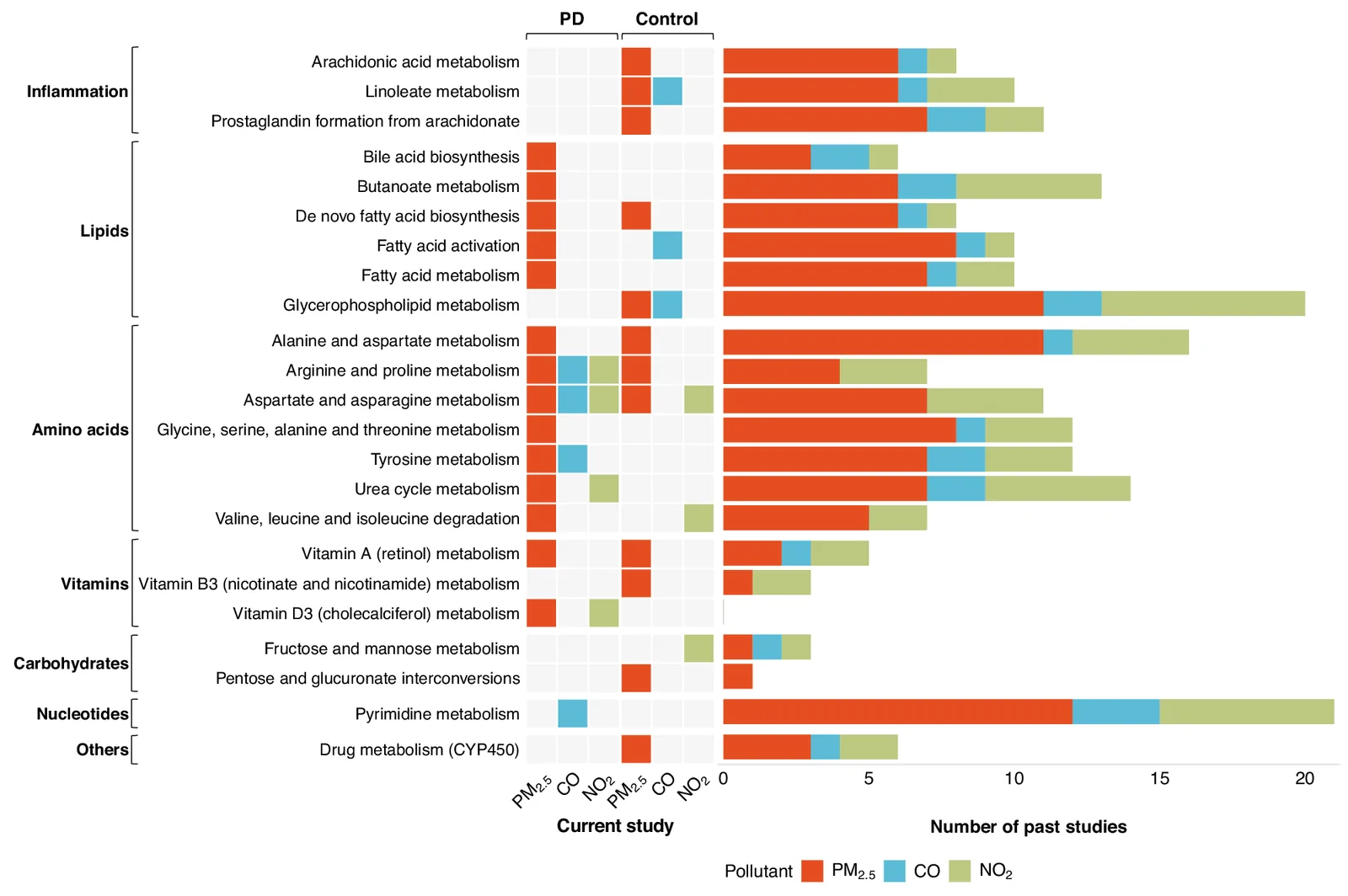
Metabolic pathways associated with air pollution exposure in Parkinson’s disease and in prior studies. Heatmap of metabolic pathway enrichment from the current study, showing associations with PM_2.5_ (orange), carbon monoxide (CO, blue), and nitrogen dioxide (NO_2_, green) in PD patients (left three columns) and controls (right three columns), for the 23 pathways enriched by ≥5 metabolites significant at *p* < 0.05 with consistent direction across PEG1 and PEG2 waves. Each cell is either filled with the corresponding pollutant color (significant pathway enrichment, *p* < 0.05) or left white (non-significant, *p* ≥ 0.05). Pathways are grouped on the y-axis by biological category (inflammation, lipids, amino acids, vitamins, carbohydrates, nucleotides, and others). Stacked bar chart summarizing pathway associations reported across 29 prior serum or plasma metabolomics studies of air pollution. The x-axis shows the cumulative number of past studies reporting each pathway, with bars segmented by pollutant: PM_2.5_, CO, and NO_2_. PD Parkinson’s disease, PEG Parkinson’s Environment and Gene, PM_2.5_ particulate matter ≤2.5 µm.

**Fig. 5:**
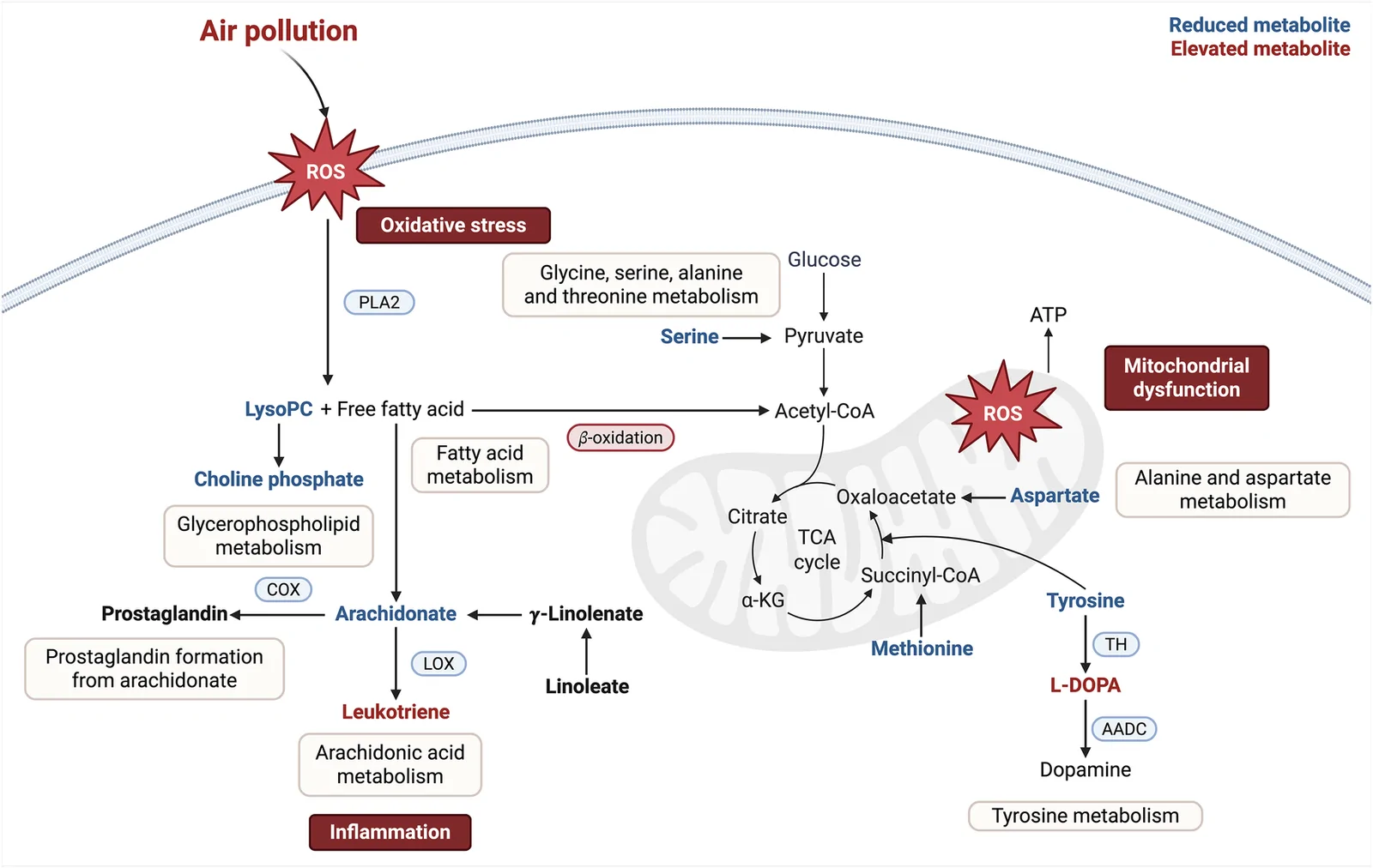
Potential molecular mechanisms linking air pollution exposure to Parkinson’s disease. Schematic summarizing pathways through which air pollution may contribute to PD pathobiology, integrating findings from the current study. Air pollution exposure generates reactive oxygen species (ROS), driving oxidative stress at the cell membrane and within mitochondria. Membrane phospholipid remodeling via phospholipase A2 (PLA2) releases lysophosphatidylcholines (LysoPC), choline phosphate, and free fatty acids, including arachidonate, which is further metabolized by cyclooxygenase (COX) to prostaglandins and by lipoxygenase (LOX) to leukotrienes, promoting inflammation. Mitochondrial dysfunction is reflected by altered amino acid inputs to the tricarboxylic acid (TCA) cycle and by reduced adenosine triphosphate (ATP) production. Tyrosine metabolism connects to dopamine biosynthesis: tyrosine is converted to 3,4-dihydroxyphenylalanine (L-DOPA) by tyrosine hydroxylase (TH) and then to dopamine by aromatic L-amino acid decarboxylase (AADC). Metabolites measured in PD patients in this study are highlighted in color: those negatively associated with air pollution exposure (decreased with higher exposure) are shown in blue, and those positively associated (increased with higher exposure) are shown in red. PD Parkinson’s disease, α-KG α-ketoglutarate.

Using untargeted serum metabolomics with liquid chromatography-high-resolution mass spectrometry (LC-HRMS), we identified 101 annotated metabolites and 23 metabolic pathways associated with air pollution, specifically PM_2.5_ and TRAP (measured by CO and NO_2_), in controls and PD patients. The metabolic profiles we observed in controls aligned well with serum and plasma findings from 29 previous air pollution metabolomics studies (Figs. 3–4 and Supplementary Data 2), supporting the validity of our approach and providing a reference against which to compare responses in PD patients. As summarized in Fig. 5, air pollution broadly disrupted inflammation-related lipid metabolism (reduced arachidonate; elevated leukotriene) and amino acid metabolism, while PD patients showed additional disruptions extending to mitochondrial dysfunction (reduced aspartate and serine) and dopamine biosynthesis (reduced tyrosine).

We identified disruptions in inflammation-related lipids with air pollution exposure in controls and PD patients. PM_2.5_ represents a complex mixture of pollutants, including PAHs^11,12^, which can enter the body via inhalation and reach the bloodstream, contributing to systemic inflammation^6–8^. These pollutants also generate reactive oxygen species (ROS)^13^, which target cell membranes, induce lipid peroxidation, and release polyunsaturated fatty acids (PUFAs), such as linoleate and arachidonate^14^. The multiple carbon-carbon double bonds in these PUFAs make them highly susceptible to oxidation by oxygenase^15^. In our study, PM_2.5_ exposure was associated with reduced arachidonate in both groups, reduced linoleate and gamma-linolenate in controls, and elevated leukotriene C4 in PD patients, aligning with findings from previous studies^16–20^. Arachidonate and linoleate serve as substrates for lipoxygenase enzymes expressed by immune cells, leading to the production of major inflammatory mediators, including prostaglandins and leukotrienes^21^. Identification of alterations in linoleate metabolism, arachidonic acid metabolism, and prostaglandin formation pathways in controls further suggests that air pollution disrupts inflammatory signaling and the antioxidant-oxidant balance in our study population. Importantly, these inflammatory pathways were also enriched in previous air pollution-metabolomics studies conducted across diverse populations^3,16,18,20,22–30^, including pregnant women, children, and young adults, suggesting that inflammation likely represents a typical metabolomic response to air pollution.

In PD patients, however, the elevated leukotriene C4 with PM_2.5_ exposure suggests a more pronounced pro-inflammatory lipid shift on top of this general response. Evidence for leukotriene involvement in PD is more limited than in Alzheimer’s disease^31^, but rodent models and mechanistic studies suggest that leukotrienes trigger microglia and astrocyte activation, leading to neuroinflammation and oxidative stress^32,33^. This pro-inflammatory shift is also consistent with the concurrent reduction in arachidonate, the fatty acid from which leukotrienes are made: rather than accumulating, arachidonate appears to be channeled forward into inflammatory mediators like leukotriene C4 (Fig. 5).

Beyond inflammation, membrane phospholipid metabolites were also altered with air pollution exposure in controls and PD patients. Choline phosphate, a precursor for phosphatidylcholine biosynthesis, was reduced with PM_2.5_ in both groups, and glycerophospholipid metabolism was enriched at the pathway level with PM_2.5_ and CO in controls. In patients, LysoPC(16:0) was also reduced with PM_2.5_, extending the phospholipid response beyond what was observed in controls. Phosphatidylcholine precursors and lysophosphatidylcholines are central to membrane integrity, vesicular dopamine packaging, and synaptic function^34^. Their depletion with air pollution exposure suggests altered membrane phospholipid remodeling, which has been implicated in alpha-synuclein aggregation and dopaminergic neurodegeneration^35^.

Multiple amino acid metabolism pathways were enriched with air pollution exposure in controls and PD patients, including alanine and aspartate metabolism. In PD patients, glycine, serine, alanine and threonine metabolism was additionally enriched, extending the amino acid response beyond what was observed in controls. At the metabolite level, aspartate, serine, and methionine were reduced with PM_2.5_ and NO_2_ in PD patients (Supplementary Data 1). These amino acids serve as substrates for the tricarboxylic acid (TCA) cycle; aspartate is converted to oxaloacetate, serine is converted to pyruvate, and methionine enters the cycle via succinyl-CoA (Fig. 5). Their depletion may therefore reduce TCA cycle flux, impair ATP production, and weaken antioxidant defenses—potentially forcing greater reliance on fatty acid beta-oxidation^36^, a less efficient energy source that generates more ROS and may exacerbate the mitochondrial dysfunction already characteristic of PD.

Tyrosine metabolism was enriched in PD patients, with tyrosine negatively associated with air pollution exposure. Tyrosine is converted into L-DOPA by tyrosine hydroxylase and subsequently decarboxylated into dopamine (Fig. 5), the neurotransmitter essential for controlling movement and one that is progressively lost in PD. Notably, while tyrosine was reduced, its direct product L-DOPA was elevated with PM_2.5_ exposure, and this association persisted after adjustment for LED, indicating that the signal is not attributable to levodopa medication intake. The directional concordance, depleted substrate (tyrosine) alongside elevated product (L-DOPA), suggests altered flux through the dopamine biosynthesis pathway, potentially reflecting compensatory tyrosine hydroxylase activation under air pollution-induced dopaminergic stress. Lower tyrosine levels have also been linked to severe depressive symptoms in our PD patients^37^, suggesting that air pollution-induced tyrosine reduction may contribute to disease phenotypes such as depression by impairing dopamine biosynthesis.

To date, our study is the first untargeted high-resolution metabolomics (HRM) study examining air pollution in the context of PD. However, several limitations should be considered. The unequal sample sizes for PD patients and controls constrained statistical power for control-only and study wave-stratified analyses; candidate associations should therefore be considered hypothesis-generating, and effect-size estimates for any single (even replicated) metabolite should be interpreted cautiously. We did not perform gender-stratified MWAS due to limited within-subgroup statistical power; gender-specific metabolic responses to air pollution warrant dedicated investigation in larger cohorts. Lifestyle factors such as smoking and diet were not associated with air pollution exposure in our cohort and therefore do not meet the definition of a confounder in this setting; they were not included as covariates, as adjustment for non-confounders does not reduce bias and may inflate variance. Residual confounding by unmeasured factors cannot be ruled out.

As PEG participants reside in a relatively high-pollution region of central California, we may have identified some metabolic disturbances specific to high-exposure settings. External replication in another geographical area with a different exposure range would therefore be of interest. Our cohort was also predominantly non-Hispanic White, and findings may not directly apply to populations with different demographic composition. However, the overlap with prior studies in demographically diverse populations strengthens our confidence that the metabolic features we identified are robustly linked to air pollution. Model-based exposure estimates did not account for personal behaviors, time-activity patterns, or indoor exposures, but this nondifferential misclassification likely biases effect estimates toward the null. Our study focuses on outdoor ambient air pollution, the exposure metric most relevant for regulatory action.

Untargeted LC-HRMS provides high-confidence putative annotations based on m/z, retention time, and feature correlation structures, but approximately 40% of the features had no match in current databases, with some matching multiple possible compounds. As reference libraries (e.g., HMDB, KEGG) continue to expand, more features will likely be annotated in future studies. Finally, our metabolomic measurements were based on a single blood draw, providing only a snapshot in time. Future longitudinal human studies, in vitro toxicological systems, animal models, and transcriptomic integration will help determine whether the metabolites identified here are causally related to PD pathomechanisms or clinical progression, or may reliably serve as exposure-response biomarkers.

Our untargeted metabolomics study identifies candidate molecular mechanisms linking air pollution (PM_2.5_ and TRAP measured by CO and NO_2_) to PD, highlighting metabolic features and pathways involved in inflammation, oxidative stress, and mitochondrial dysfunction. The consistency of metabolic disruptions we observed in controls with findings from previous air pollution metabolomics studies supports the validity of our results. These hypothesis-generating findings strengthen the biological plausibility for air pollution playing a role in PD and provide molecular-level evidence that complements traditional epidemiological findings. The potential of PM_2.5_ and TRAP to disrupt metabolic responses associated with PD underscores the need for longitudinal studies with repeated measures of air pollution exposure, metabolomics, and motor and non-motor symptoms to determine whether pollution-induced metabolic changes precede or coincide with disease onset or progression.

## Methods

### Study population

We analyzed metabolomic profiles in 616 PD patients and 271 population-based controls from the PEG study, conducted in Fresno, Kern, and Tulare counties in central California. Participants were recruited in two waves: PEG1 (2000–2007) and PEG2 (2010–2017). All individuals with available serum samples for metabolomics analysis were included.

PD patients were eligible if they had resided in California for at least five years and had received a PD diagnosis within the past three (PEG1) or five years (PEG2), with an average time since diagnosis of 3 years (SD = 2.6). Recruitment sources included local clinics, neurologists, medical groups, radio advertisements, and the California PD registry. All participants underwent in-person neurologic examinations conducted by University of California, Los Angeles (UCLA) movement disorder specialists, who confirmed idiopathic PD based on clinical characteristics. At the time of serum collection, 444 (72%) of the PD patients in our analytic sample were taking levodopa (median LED = 400 mg/day, interquartile range [IQR] = 225).

Controls were randomly selected from Medicare rolls or property tax assessor records listings of residential parcels. Additional inclusion criteria for controls included being at least 35 years of age, having resided in California for five years or more, currently residing in one of the three counties, and not having received a diagnosis of Parkinsonism. Further details about recruitment procedures have been published elsewhere^38^.

The study was conducted in accordance with the Declaration of Helsinki, and all methods were carried out in accordance with relevant guidelines and regulations. The study protocol was approved by the Institutional Review Board of the University of California, Los Angeles (IRB#21-000256 and IRB#11-001530). Written informed consent was obtained from all participants prior to participation.

### Air pollution exposure assessment

We used established air pollution models to estimate annual average pollutant concentrations at participants’ geocoded residential locations for each year up to the year of PD diagnosis for cases or the interview year for controls (the latest possible year was 2016). We focused our exposure assessment on the period from 1996 to 2016 to align with the timeframe of serum sample collection for metabolomics profiling.

The main exposures in this study were PM_2.5_, modeled using the CTM, and local TRAP, measured by CO using the CALINE4. For PM_2.5_, we applied a fine-resolution geospatial model providing validated 1 km resolution exposure estimates across North America^39^. This model integrates GEOS-Chem CTM outputs, satellite aerosol optical depth (AOD) observations, and ground-based PM_2.5_ measurements using geographically weighted regression. For local TRAP, the CALINE4 dispersion model estimated CO concentrations by summing up vehicle exhaust emissions dispersed from roadway links within 1.5 km of geocoded residential addresses using local traffic and meteorological data^40,41^.

We also examined additional exposures, including PM_2.5_ and regional TRAP, measured by NO_2_, both modeled using the LUR approach for the same 1996–2016 period^42^. PM₂.₅ estimates were derived from fixed-site government-regulatory monitoring data and Google Street View data, while NO_2_ estimates additionally incorporated research-based saturation observations. The LUR models provided 100 m spatial resolution and incorporated inputs such as daily vehicle miles traveled, remote sensing data (AOD and NO₂), biweekly vegetation indices, daily weather data, and land-use/land-cover features.

For brevity, we refer to pollutants in the format *pollutant (modeling approach)* throughout the text—for example, PM_2.5_ (CTM), CO (CALINE4), PM_2.5_ (LUR), and NO_2_ (LUR). In this study, the CTM- and CALINE4-based models were prioritized based on their stronger validation performance (*R*^2^ = 0.86 for PM_2.5_ [CTM] and 0.92 for CO [CALINE4]) compared with the LUR-based models (*R*^2^ = 0.65 for PM_2.5_ [LUR] and 0.84 for NO_2_ [LUR])^39,42,43^, and their prior use in air pollution-PD epidemiologic studies conducted in California and globally^2,44^.

We focused on exposure windows of 1, 3, and 5 years to balance etiologic relevance—given the long prodromal phase of PD—with the more proximal physiological states reflected in serum metabolomic profiles. We calculated each average using the calendar years preceding the year of serum sample collection and examined per IQR increases in pollutant levels. Since serum sample collection began in 2001, the earliest exposure window analyzed was 1996–2001 for the 5-year average. Participants for whom we had data available for at least 60% of the exposure period were included in the analysis, and any remaining missing data (up to 40% of the period) were imputed by averaging concentrations from adjacent years (4 patients and 3 controls). A small proportion of participants (0.1–3%) had imputed values, and mean exposure levels were comparable between those with and without imputed data based on t-tests (*p* = 0.4–1.0), suggesting minimal exposure misclassification due to imputation.

### High-resolution metabolomics (HRM)

Serum samples were collected during baseline study visits^45^, centrifuged, aliquoted, and stored at −80 °C at UCLA before being shipped on dry ice to Emory University, where they were stored at −80 °C until analysis. Samples were prepared by mixing with ice-cold acetonitrile (2:1 acetonitrile:serum), incubated on ice for 30 min, and centrifuged to remove precipitated protein; the supernatant was transferred to autosampler vials^5^. All sample extracts were analyzed in triplicate using a dual-column, dual-polarity LC-HRMS platform combining hydrophilic interaction chromatography (HILIC) with positive electrospray ionization (ESI+) and C18 chromatography with negative ESI (ESI−), following a standard HRM workflow^5,37,45^.

Samples were processed across two analytical runs, each comprising 15 sequential batches of randomly sorted samples. Two types of quality control (QC) samples were included to evaluate analytical performance and to enable batch-effect correction: NIST Standard Reference Material 1950 (pooled human plasma) was analyzed at the beginning and end of each analytical run^46^, and a commercial pooled-plasma reference (Q-Std) was analyzed at the start, middle, and end of every batch. Per-batch quality was evaluated using XCMS-based diagnostics for the number of detected features, missing-value frequency, mass accuracy (<5 ppm), Pearson correlation among technical replicates (>0.9), and average coefficient of variation (CV) of feature intensities among replicates (<30%). Batches that failed any criterion were re-analyzed^5^.

Raw spectra were processed and aligned across the two analytical runs using the *apLCMS* R package^47^, with retention-time tolerances of 38.246 s for the HILIC and 37.016 s for the C18 and an *m/z* tolerance of 1 × 10^−5^, yielding 2913 HILIC features and 2222 C18 features. Features were retained for downstream analysis if (1) the median CV among technical replicates was <30%, (2) the Pearson correlation among replicates was >0.9, and (3) the feature was detected in >50% of all study samples, resulting in a filtered feature matrix of 2716 HILIC and 2046 C18 features. Missing values were imputed using the feature-wise non-zero minimum, and intensities were log_2_-transformed and median-normalized.

Batch effects were removed in sequential ComBat passes implemented in the *sva* R package^48^, beginning with the LC-HRMS run/batch as the known batch indicator. After exclusion of pooled-plasma QC samples, principal-component analysis (PCA) revealed residual clustering not explained by run/batch alone; additional ComBat corrections were therefore applied using indicators for these clustering patterns to further reduce non-biological variation^5^. Effectiveness of correction was evaluated by visualizing per-sample mean intensities and principal-component embeddings before and after each step, with pooled-QC samples used as a stability anchor; PCA before and after batch correction has been documented in detail for this identical pipeline (Supplementary Figs 5–7 of Paul et al.^5^).

### Statistical analysis

To identify metabolite features associated with air pollution exposure, we performed an MWAS in PD patients using empirical Bayes linear regression models. The model estimated log_2_ fold changes (log_2_FC) per IQR increase in air pollution, adjusting for age, gender, race/ethnicity (white vs. non-white), education (years), sample collection year, and study wave (PEG1 vs. PEG2). We classified significant features into three confidence tiers: (1) false discovery rate (FDR) < 0.05 for the most stringent findings; (2) FDR < 0.10 for moderately supported findings; and (3) nominal *p* < 0.05 with replicated (same) direction of effect estimates in both PEG1 and PEG2 for exploratory findings (Supplementary Table 1). Requiring a consistent effect estimate direction across PEG1 and PEG2 strengthens the credibility of findings. High correlations of effect estimates across study waves support the reproducibility of associations and help prioritize candidate metabolites for downstream analysis (Supplementary Fig 1).

We additionally performed a full MWAS in control participants as an independent analysis. This allowed us to assess whether the associations observed in PD patients were also present in individuals without PD and to check whether control results in general aligned with prior findings from air pollution-metabolomics studies. Comparator studies were restricted to those matching our analysis on biosample (serum or plasma) and pollutant (PM_2.5_, CO, or NO_2_). Comparing the two sets of results enabled us to explore metabolic responses to air pollution that were observed in PD patients but not in controls or vice versa. We also repeated analyses using alternative exposure assessment models (PM_2.5_ and NO_2_ from LUR) to evaluate robustness across different air pollution modeling approaches.

Pathway analyses were performed using the *mummichog* approach in *MetaboAnalystR*^49^, using exploratory features (*p* < 0.05 with replication) as input, with a mass tolerance of 10 ppm and mixed ion mode. Pathways were selected if they included ≥5 significant features and showed overrepresentation with *p* < 0.05.

We annotated metabolite features (*p* < 0.05 with replication) that were also enriched in a metabolic pathway (*p* < 0.05). This approach helped minimize false positives while allowing the use of a less statistically conservative threshold for MWAS to guide exploratory analyses. Annotation was performed using three confidence tiers^50^: (1) features were matched to a database of authenticated chemical standards identified by the Emory laboratory using MS/MS and authentic standards, as these offer the highest annotation confidence^51,52^. The error tolerance was set to 10 ppm for m/z and 30 s for retention time; (2) *mummichog* was used to predict functional activity and to annotate features based on metabolic pathways and networks using predicted ions and pathway associations^53^; and (3) *xMSannotator* matched m/z values for adducts (positive/negative ESI mode) to the Human Metabolome Database with a 10 ppm mass error threshold, leveraging intensity and retention time correlations^54^. Confidence scores (0–3) were assigned based on a multilevel scoring algorithm, and only annotations with a score ≥2 were included.

As a sensitivity analysis, we re-ran the MWAS in PD patients with additional adjustment for LED to assess whether observed air pollution-metabolite associations could be attributable to levodopa medication-derived contributions to circulating metabolites.

## Supplementary information

supplementary_information

supplementary_data2

supplementary_data1

## Data Availability

The metabolomics data used in this study are publicly available on Metabolomics Workbench (Project ID: PR001964, https://doi.org/10.21228/M8VD96).

## Code availability

Analysis code is publicly available at https://github.com/dayoonkwon/PEGAirPollution.
