# Supplementary material for: Untargeted serum metabolomics and air pollution in Parkinson’s disease
Source: NPJ Parkinsons Dis. 2026 Jul 1;12:244. doi: 10.1038/s41531-026-01451-3 (PMC13646296; doi:10.1038/s41531-026-01451-3)
Supplement: Supplementary file 1 [file 41531_2026_1451_MOESM1_ESM.pdf]

Supplementary Information

Supplementary Tables 1

Supplementary Table 1. Overview of study design, population, exposure assessment, metabolomics platform, statistical analysis, and metabolite annotation strategy in the Parkinson's Environment and Gene (PEG) study. 1

Supplementary Table 2. Descriptive statistics of estimated air pollution concentrations over the 1-, 3-, and 5-year periods prior to serum sample collection in the PEG study. 2

Supplementary Table 3. Counts of serum metabolite features significantly associated with air pollution exposure in Parkinson's disease patients, by exposure model, exposure window, ionization mode, and statistical-significance threshold. 3

Supplementary Table 4. Sensitivity analysis comparing air pollution–metabolite associations in Parkinson's disease patients before and after additional adjustment for levodopa equivalent daily dose (LED). 4

Supplementary Table 5. Counts of serum metabolite features significantly associated with air pollution exposure in controls, by exposure model, exposure window, ionization mode, and statistical-significance threshold. 5

Supplementary Table 6. Counts of replicated metabolite features (nominal p<0.05 with consistent direction across PEG1 and PEG2) classified by sharing between Parkinson's disease patients and controls. 6

Supplementary Figures 7

Supplementary Figure 1. Replication of metabolite–air pollution associations in PEG1 and PEG2 among Parkinson's disease patients. 7

Supplementary Figure 2. Spearman correlations between 1-, 3-, and 5-year average air pollution exposures prior to serum collection. 8

Supplementary Figure 3. Comparison of air pollution-associated metabolomic changes in PD patients and controls. 9

Supplementary Figure 4. Metabolomic features associated with air pollution exposure in controls. 10

Supplementary Figure 5. Metabolic pathway enrichment by air pollution exposure across pollutants and exposure windows in PD patients and controls. 11

Supplementary Tables

Supplementary Table 1. Overview of study design, population, exposure assessment, metabolomics platform, statistical analysis, and metabolite annotation strategy in the Parkinson's Environment and Gene (PEG) study.

Category	Parameter	Value/description
**Study design**	Design	Population-based case-control
	Cohort	Parkinson's Environment and Gene (PEG) study
	Region	Fresno, Kern, and Tulare counties, central California
	Recruitment waves	PEG1 (2000-2007); PEG2 (2010-2017)
	Sample size	616 PD patients; 271 controls
**Population**	PD eligibility	Idiopathic PD, neurologist-confirmed; California residency ≥5 years; PD diagnosis ≤3 (PEG1) or ≤5 (PEG2) years
	Control eligibility	Age ≥35; California residency ≥5 years; current residence in 3 study counties; no Parkinsonism diagnosis
	PD duration (mean ± SD)	3.0 ± 2.6 years
	Age at sample collection	PD: 70 ± 10 y; Controls: 66 ± 13 y
**Exposure**	Pollutants	$PM_{2.5}$, CO, NO_2
	Modeling approaches	(1) CTM for $PM_{2.5}$; (2) CALINE4 for CO; (3) LUR for $PM_{2.5}$ and NO_2
	Spatial resolution	CTM: 1km; CALINE4: residence-level, 1.5km buffer; LUR: 100m
	Validation R^2	$PM_{2.5}$ (CTM): 0.86; CO (CALINE4): 0.92; $PM_{2.5}$ (LUR): 0.65; NO_2 (LUR): 0.84
	Exposure windows	1-, 3-, and 5-year averages prior to serum collection
	Period assessed	1996-2016
**Metabolomics**	Sample type	Serum
	Platform	LC-HRMS
	Columns/ionization	HILIC + positive ESI; C18 + negative ESI; triplicate analysis
	QC	NIST 1950 reference; pooled plasma per batch (n=40); CV<30%; mass accuracy <5ppm
	Features detected	2,716 HILIC + 2,046 C18 (after filtering)
	Preprocessing	Log_2 transform → quantile normalization → ComBat by run batch → ComBat by PCA-cluster (*sva* package)
**Statistical analysis**	MWAS model	Empirical Bayes linear regression (*limma*)
	Outcome	Log_2 fold change per IQR increase in pollutant
	Adjusted covariates	Age, gender, race/ethnicity, education, sample collection year, study wave
	Statistical significance criteria	Tier 1: FDR<0.05
		Tier 2: FDR<0.10
		Tier 3: nominal p<0.05 with consistent effect direction in both PEG1 and PEG2 (replication)
	Pathway analysis	*MetaboAnalystR* with mixed ion mode, 10ppm tolerance
	Pathway inclusion	≥5 air pollution-associated metabolites + p<0.05 enrichment
**Annotation**	Tier 1	Authenticated chemical standards (Emory laboratory, MS/MS + RT, 10ppm m/z, 30s RT)
	Tier 2	*Mummichog* network/pathway-based prediction
	Tier 3	*xMSannotator*: HMDB matching (10ppm); confidence scores 2-3 retained
**Validation strategy**	Internal	Replication across PEG1 and PEG2 waves
	External	Comparison with 29 prior serum/plasma air-pollution metabolomics studies

Supplementary Table 2. Descriptive statistics of estimated air pollution concentrations over the 1-, 3-, and 5-year periods prior to serum sample collection in the PEG study.

Pollutant	Unit	Model	Mean	SD	Min	Q1	Median	Q3	Max	IQR
**1-year average exposure**										
$PM_{2.5}$	μg/m^3	CTM	12.7	3.6	1.0	10.3	12.4	15.5	21.2	5.2
CO	ppb	CALINE4	3.7	8.9	1.0×10^{-2}	0.4	1.2	3.0	94.0	2.6
$PM_{2.5}$	μg/m^3	LUR	13.6	2.1	5.0	12.5	13.9	14.9	18.9	2.4
NO_2	μg/m^3	LUR	9.8	2.2	2.3	8.3	9.7	11.5	17.6	3.2
**3-year average exposure**										
$PM_{2.5}$	μg/m^3	CTM	13.0	3.7	1.1	10.4	12.6	16.0	21.4	5.5
CO	ppb	CALINE4	4.2	10.4	1.0×10^{-2}	0.5	1.3	3.7	114.6	3.3
$PM_{2.5}$	μg/m^3	LUR	13.9	2.1	5.4	12.8	14.2	15.2	18.2	2.4
NO2	μg/m^3	LUR	10.1	2.3	2.5	8.5	10.0	11.8	20.1	3.3
**5-year average exposure**										
$PM_{2.5}$	μg/m^3	CTM	13.2	3.6	1.1	10.6	13.2	16.3	22.1	5.7
CO	ppb	CALINE4	4.6	11.3	1.0×10^{-2}	0.6	1.5	4.1	120.7	3.5
$PM_{2.5}$	μg/m^3	LUR	14.0	2.0	5.4	13.0	14.5	15.4	18.2	2.3
NO_2	μg/m^3	LUR	10.5	2.3	2.7	8.9	10.4	12.1	21.2	3.2

CALINE4, California Line Source Dispersion Model, version 4; CO, carbon monoxide; CTM, chemical transport model; IQR, interquartile range; LUR, land use regression; Max, maximum; Min, minimum; NO_2, nitrogen dioxide; $PM_{2.5}$, particulate matter ≤2.5μm; ppb, parts per billion; Q1, first quartile; Q3, third quartile; SD, standard deviation.

Supplementary Table 3. Counts of serum metabolite features significantly associated with air pollution exposure in Parkinson's disease patients, by exposure model, exposure window, ionization mode, and statistical-significance threshold.

		HILIC Positive (n = 2716)				C18 Negative (n = 2046)			
**Pollutant**	**Year**	**FDR<0.05**	**FDR<0.10**	**p<0.05**	**Replication[a]**	**FDR<0.05**	**FDR<0.10**	**p<0.05**	**Replication[a]**
**$PM_{2.5}$**	1-year	0	0	169	154	8	43	268	255
**(CTM)**	3-year	0	0	170	162	5	36	254	245
	5-year	0	0	145	138	14	29	230	221
**CO**	1-year	31	55	252	190	0	0	57	45
**(CALINE4)**	3-year	30	66	266	181	0	0	54	42
	5-year	29	62	261	167	0	0	59	46
**$PM_{2.5}$**	1-year	0	0	104	100	0	0	152	151
**(LUR)**	3-year	0	0	107	103	0	0	122	119
	5-year	1	1	113	106	0	0	119	114
**NO_2**	1-year	0	4	196	178	0	1	145	135
**(LUR)**	3-year	0	0	156	144	0	0	140	137
	5-year	0	0	142	136	0	0	117	115

Significance thresholds shown: FDR <0.05, FDR <0.10, nominal p<0.05, and nominal p<0.05 with replication.

[a]Number of metabolite features showing nominal statistical significance (p<0.05) in both the PEG1 and PEG2 study waves with consistent direction of effect estimates.

C18, octadecyl chromatography (negative ESI); CALINE4, California Line Source Dispersion Model, version 4; CO, carbon monoxide; CTM, chemical transport model; ESI, electrospray ionization; FDR, false discovery rate; HILIC, hydrophilic interaction chromatography (positive ESI); LUR, land use regression; NO_2, nitrogen dioxide; PEG, Parkinson's Environment and Gene; $PM_{2.5}$, particulate matter ≤2.5μm.

Supplementary Table 4. Sensitivity analysis comparing air pollution–metabolite associations in Parkinson's disease patients before and after additional adjustment for levodopa equivalent daily dose (LED).

				Main analysis		Sensitivity analysis	
**Metabolite**	**Pollutant (model)**	**Exposure window**	**Mode**	**Log_2FC[a]**	**p-value**	**Log_2FC[b]**	**p-value**
3,4-Dihydroxyphenylalanine	$PM_{2.5}$ (CTM)	1-yr	C18	0.258	0.002	0.254	0.002
3,4-Dihydroxyphenylalanine	$PM_{2.5}$ (CTM)	3-yr	C18	0.216	0.018	0.211	0.021
3,4-Dihydroxyphenylalanine	$PM_{2.5}$ (CTM)	5-yr	C18	0.208	0.024	0.202	0.029
3,4-Dihydroxyphenylalanine	$PM_{2.5}$ (LUR)	1-yr	C18	0.141	0.036	0.137	0.042
Arachidonate	$PM_{2.5}$ (CTM)	1-yr	C18	-0.203	0.002	-0.197	0.003
Arachidonate	$PM_{2.5}$ (CTM)	3-yr	C18	-0.193	0.007	-0.185	0.009
Arachidonate	$PM_{2.5}$ (CTM)	5-yr	C18	-0.187	0.009	-0.178	0.013
Arachidonate	$PM_{2.5}$ (LUR)	1-yr	C18	-0.121	0.020	-0.115	0.027
Aspartate	$PM_{2.5}$ (CTM)	1-yr	HILIC	-0.199	0.004	-0.199	0.004
Aspartate	$PM_{2.5}$ (CTM)	3-yr	HILIC	-0.285	1.4×10^{-4}	-0.285	1.5×10^{-4}
Aspartate	$PM_{2.5}$ (CTM)	5-yr	HILIC	-0.276	2.4×10^{-4}	-0.275	2.6×10^{-4}
Aspartate	NO_2 (LUR)	1-yr	HILIC	-0.166	0.026	-0.164	0.028
Aspartate	NO_2 (LUR)	3-yr	HILIC	-0.155	0.042	-0.155	0.044
Choline phosphate	CO (CALINE4)	1-yr	HILIC	-0.072	1.1×10^{-4}	-0.071	1.3×10^{-4}
Choline phosphate	CO (CALINE4)	3-yr	HILIC	-0.076	1.5×10^{-4}	-0.075	1.8×10^{-4}
Choline phosphate	CO (CALINE4)	5-yr	HILIC	-0.077	1.3×10^{-4}	-0.076	1.7×10^{-4}
Choline phosphate	$PM_{2.5}$ (CTM)	1-yr	HILIC	-0.231	0.042	-0.241	0.034
Choline phosphate	$PM_{2.5}$ (CTM)	3-yr	HILIC	-0.310	0.012	-0.324	0.008
Choline phosphate	$PM_{2.5}$ (CTM)	5-yr	HILIC	-0.308	0.013	-0.325	0.009
Leukotriene C4	$PM_{2.5}$ (CTM)	5-yr	C18	0.423	0.041	0.407	0.049
LysoPC(16:0)	$PM_{2.5}$ (LUR)	1-yr	HILIC	-0.070	0.046	-0.067	0.056
Methionine	NO_2 (LUR)	1-yr	HILIC	-0.193	0.008	-0.189	0.009
Methionine	NO_2 (LUR)	3-yr	HILIC	-0.202	0.004	-0.199	0.005
Methionine	NO_2 (LUR)	5-yr	HILIC	-0.162	0.021	-0.159	0.022
Retinoate	$PM_{2.5}$ (LUR)	1-yr	C18	-0.382	0.036	-0.391	0.032
Serine	$PM_{2.5}$ (CTM)	3-yr	HILIC	-0.102	0.021	-0.097	0.028
Serine	$PM_{2.5}$ (CTM)	5-yr	HILIC	-0.099	0.025	-0.093	0.036
Tyrosine	NO_2 (LUR)	1-yr	HILIC	-0.165	0.026	-0.168	0.025
Tyrosine	NO_2 (LUR)	3-yr	HILIC	-0.185	0.016	-0.185	0.016
Tyrosine	NO_2 (LUR)	5-yr	HILIC	-0.156	0.040	-0.156	0.039
dTDP	CO (CALINE4)	1-yr	HILIC	-0.142	9.6×10^{-5}	-0.141	1.1×10^{-4}
dTDP	CO (CALINE4)	3-yr	HILIC	-0.146	1.6×10^{-4}	-0.145	1.8×10^{-4}
dTDP	CO (CALINE4)	5-yr	HILIC	-0.146	1.9×10^{-4}	-0.145	2.1×10^{-4}

[a]Adjusted for age, gender, race/ethnicity, education, sample year, and study wave.
[b]Adusted for age, gender, race/ethnicity, education, sample year, study wave, and LED.
C18, octadecyl chromatography (negative ESI); CALINE4, California Line Source Dispersion Model, version 4; CO, carbon monoxide; CTM, chemical transport model; ESI, electrospray ionization; HILIC, hydrophilic interaction chromatography (positive ESI); LED, levodopa equivalent daily dose; log_2FC, log_2 fold change per interquartile-range increase in pollutant; LUR, land use regression; LysoPC, lysophosphatidylcholine; NO_2, nitrogen dioxide; $PM_{2.5}$, particulate matter ≤2.5μm.

Supplementary Table 5. Counts of serum metabolite features significantly associated with air pollution exposure in controls, by exposure model, exposure window, ionization mode, and statistical-significance threshold.

		HILIC Positive (n = 2716)				C18 Negative (n = 2046)			
**Pollutant**	**Year**	**FDR<0.05**	**FDR<0.10**	**p<0.05**	**Replication[a]**	**FDR<0.05**	**FDR<0.10**	**p<0.05**	**Replication[a]**
**$PM_{2.5}$**	1-year	0	0	161	121	0	0	161	122
**(CTM)**	3-year	0	0	136	97	0	0	151	109
	5-year	0	0	130	87	0	0	137	101
**CO**	1-year	0	2	120	80	1	2	72	49
**(CALINE4)**	3-year	0	1	114	76	1	1	67	48
	5-year	0	0	101	64	1	1	94	69
**$PM_{2.5}$**	1-year	0	0	140	132	0	0	167	155
**(LUR)**	3-year	0	0	131	125	0	1	166	156
	5-year	0	0	150	134	1	5	189	165
**NO_2**	1-year	0	0	105	83	0	0	50	44
**(LUR)**	3-year	0	0	110	101	0	0	62	54
	5-year	0	0	111	102	0	0	66	61

[a]Number of metabolite features showing nominal statistical significance ($p<0.05$) in both PEG1 and PEG2 with consistent direction of effect estimates.
C18, octadecyl chromatography (negative ESI); CALINE4, California Line Source Dispersion Model, version 4; CO, carbon monoxide; CTM, chemical transport model; ESI, electrospray ionization; FDR, false discovery rate; HILIC, hydrophilic interaction chromatography (positive ESI); LUR, land use regression; NO_2, nitrogen dioxide; PEG, Parkinson's Environment and Gene; $PM_{2.5}$, particulate matter ≤2.5μm.

Supplementary Table 6. Counts of replicated metabolite features (nominal p<0.05 with consistent direction across PEG1 and PEG2) classified by sharing between Parkinson's disease patients and controls.

		HILIC Positive (n = 2716)			C18 Negative (n = 2046)		
**Pollutant**	**Year**	**Both[a]**	**PD[b]**	**Control[c]**	**Both[a]**	**PD[b]**	**Control[c]**
**$PM_{2.5}$ (CTM)**	1-year	6	148	115	6	249	116
	3-year	3	159	94	6	239	103
	5-year	5	133	82	6	215	95
**CO (CALINE4)**	1-year	8	182	72	0	45	49
	3-year	7	174	69	0	42	48
	5-year	3	164	61	0	46	69
**$PM_{2.5}$ (LUR)**	1-year	7	93	125	8	143	149
	3-year	9	93	116	2	117	154
	5-year	8	98	127	2	112	163
**NO_2 (LUR)**	1-year	5	173	78	3	132	41
	3-year	4	140	97	0	137	54
	5-year	3	133	99	2	113	59

[a]Features passing p<0.05 with replication in PD patients and controls.
[b]Features passing p<0.05 with replication in PD patients only.
[c]Features passing p<0.05 with replication in controls only.
C18, octadecyl chromatography (negative ESI); CALINE4, California Line Source Dispersion Model, version 4; CO, carbon monoxide; CTM, chemical transport model; ESI, electrospray ionization; HILIC, hydrophilic interaction chromatography (positive ESI); LUR, land use regression; NO_2, nitrogen dioxide; PD, Parkinson's disease; PEG, Parkinson's Environment and Gene; $PM_{2.5}$, particulate matter ≤2.5µm.

Supplementary Figures

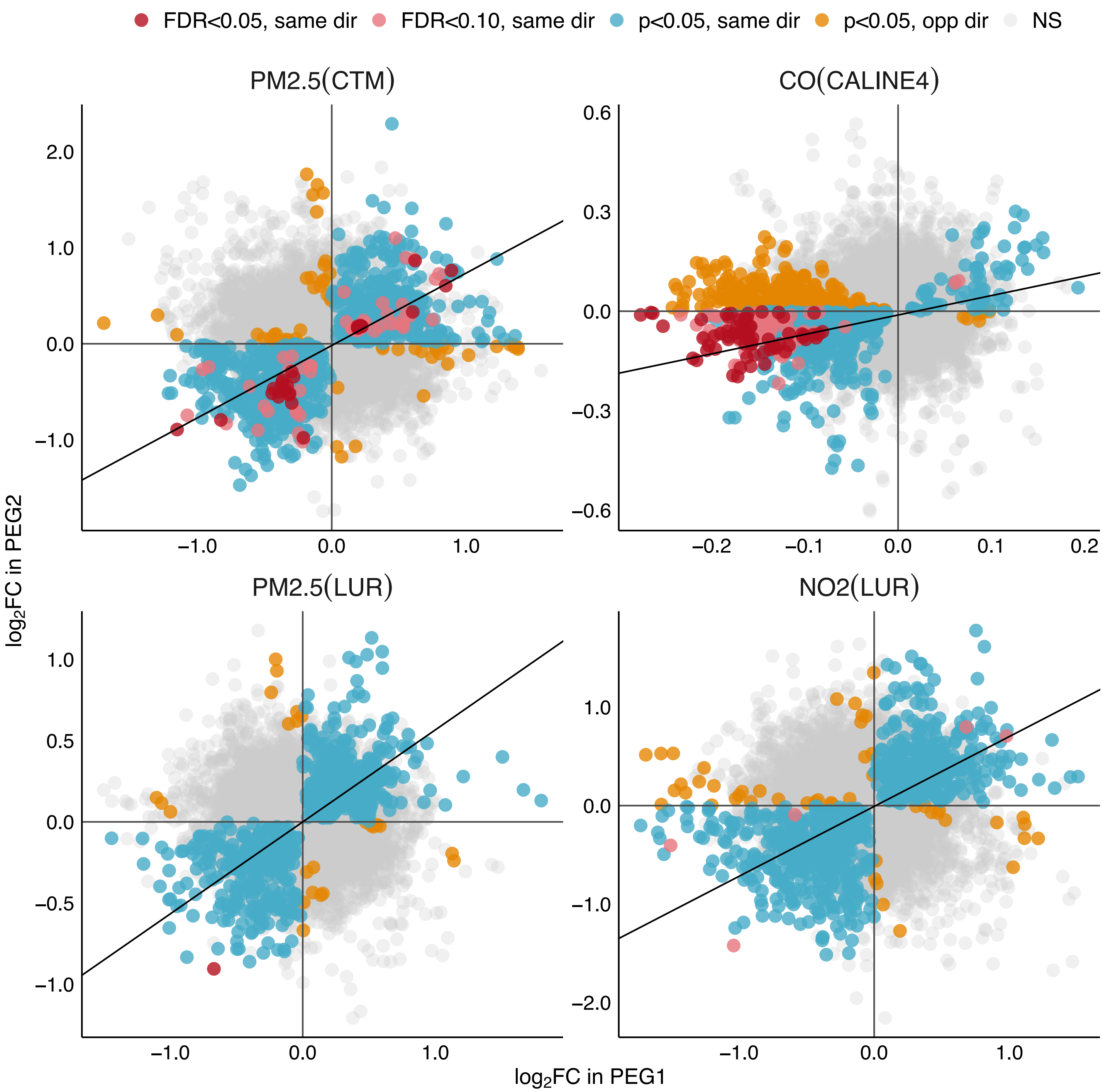

Supplementary Figure 1. Replication of metabolite–air pollution associations in PEG1 and PEG2 among Parkinson's disease patients. Scatter plots showing $\log_2$ fold change ($\log_2$FC) of metabolite features associated with air pollution exposure in the PEG1 wave (x-axis) versus the PEG2 wave (y-axis) among PD patients. Each point represents one metabolite feature. Panels show four exposure models: a) $PM_{2.5}$ estimated by a chemical transport model (CTM); b) carbon monoxide (CO) by the California Line Source Dispersion Model, version 4 (CALINE4); c) $PM_{2.5}$ by land use regression (LUR); and d) nitrogen dioxide (NO_2) by LUR. Point colors indicate statistical significance and concordance of effect-estimate direction between waves: red, false discovery rate (FDR)<0.05 with concordant direction; pink, FDR<0.10 with concordant direction; blue, nominal p<0.05 with concordant direction; yellow, nominal p<0.05 with discordant direction; grey, not significant. PD, Parkinson's disease; PEG, Parkinson's Environment and Gene; $PM_{2.5}$, particulate matter ≤2.5μm.

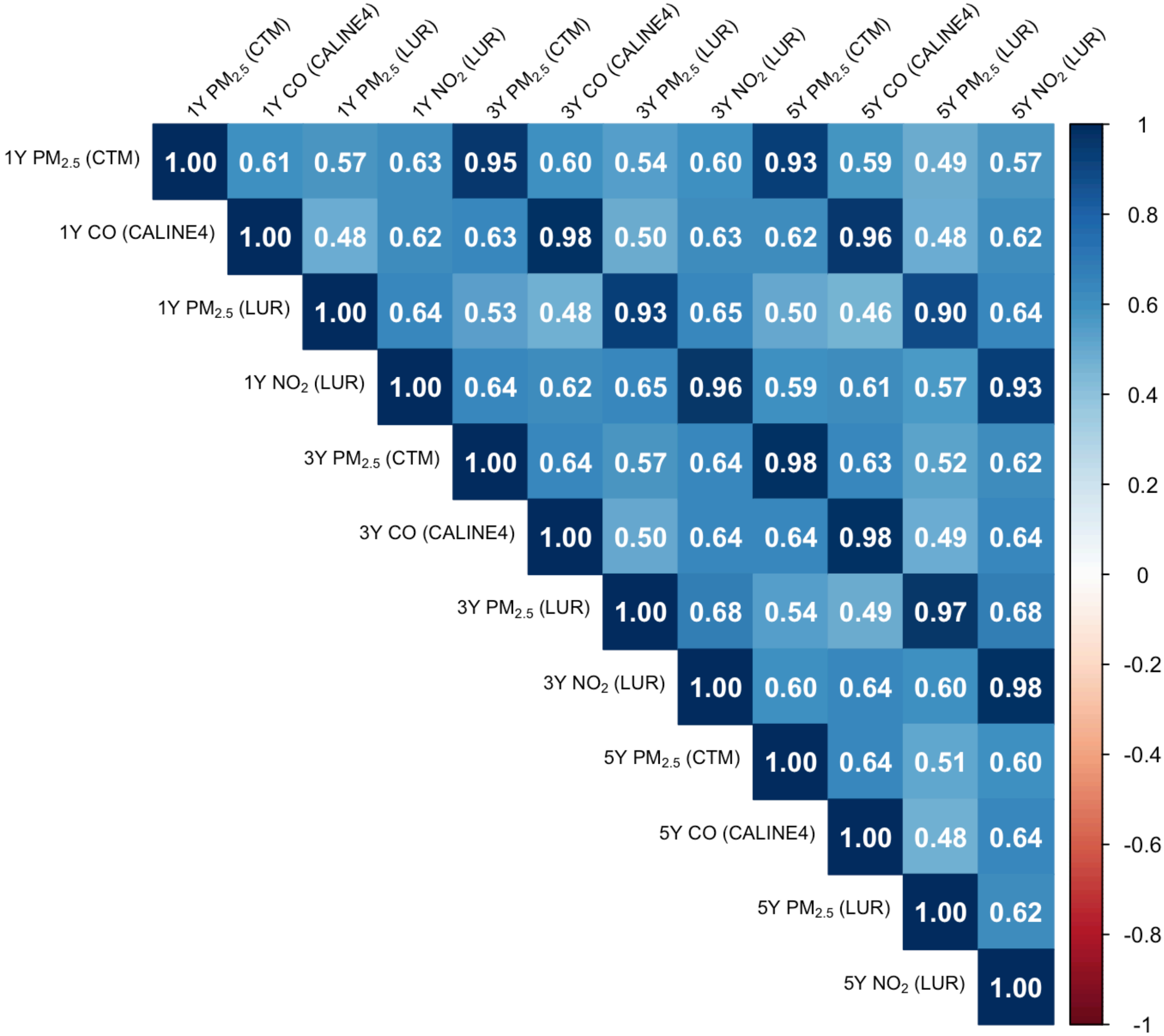

Supplementary Figure 2. Spearman correlations between 1-, 3-, and 5-year average air pollution exposures prior to serum collection. Correlation matrix showing pairwise Spearman correlation coefficients between the twelve air pollution exposure variables (four pollutant models × three averaging windows). Pollutant models are $PM_{2.5}$ estimated by a chemical transport model (CTM), carbon monoxide (CO) by the California Line Source Dispersion Model, version 4 (CALINE4), $PM_{2.5}$ by land use regression (LUR), and nitrogen dioxide (NO_2) by LUR. Averaging windows are the 1-year, 3-year, and 5-year periods prior to serum collection (labeled 1Y, 3Y, 5Y). Numeric values in each cell indicate the Spearman correlation coefficient. Cell color reflects the strength and direction of correlation: deeper blue indicates stronger positive correlations and deeper red indicates stronger negative correlations, on a continuous scale from −1 to +1. $PM_{2.5}$, particulate matter ≤2.5µm.

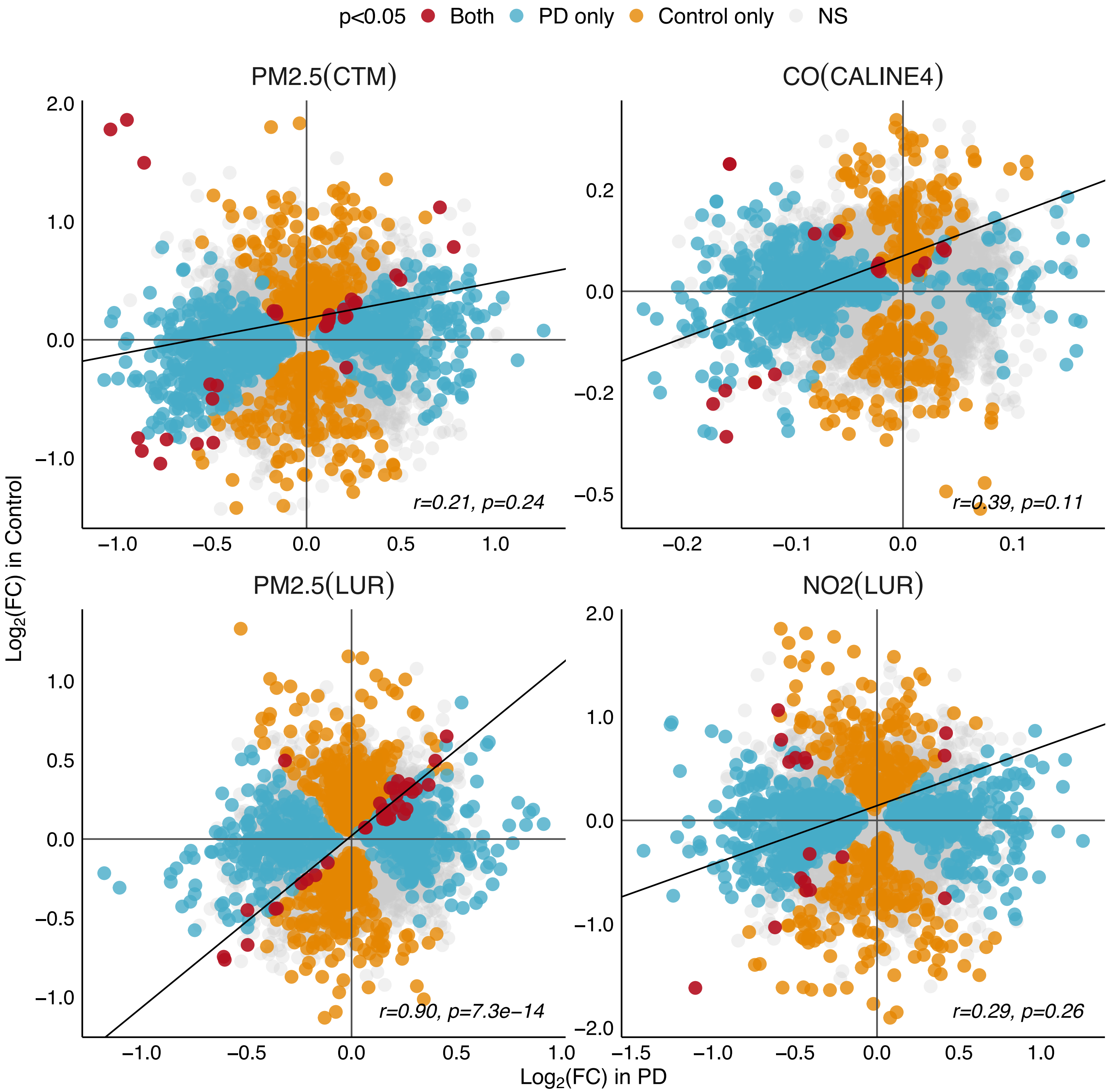

Supplementary Figure 3. Comparison of air pollution-associated metabolomic changes in PD patients and controls. Scatter plots comparing log_2 fold change (log_2FC) of metabolite features associated with air pollution exposure between PD patients (x-axis) and controls (y-axis). Each point represents one metabolite feature. Panels show four exposure models: a) $PM_{2.5}$ estimated by a chemical transport model (CTM); b) carbon monoxide (CO) by the California Line Source Dispersion Model, version 4 (CALINE4); c) $PM_{2.5}$ by land use regression (LUR); and d) nitrogen dioxide (NO_2) by LUR. Point colors indicate which group(s) the metabolite was significant in (nominal $p<0.05$ with consistent direction across PEG1 and PEG2): shared hits in both PD patients and controls (red), PD-only hits (blue), control-only hits (yellow), and non-significant features (grey). Pearson correlation coefficients (r) and corresponding p-values are reported in each panel for the shared hits. PD, Parkinson's disease; PEG, Parkinson's Environment and Gene; $PM_{2.5}$, particulate matter ≤2.5 μm.

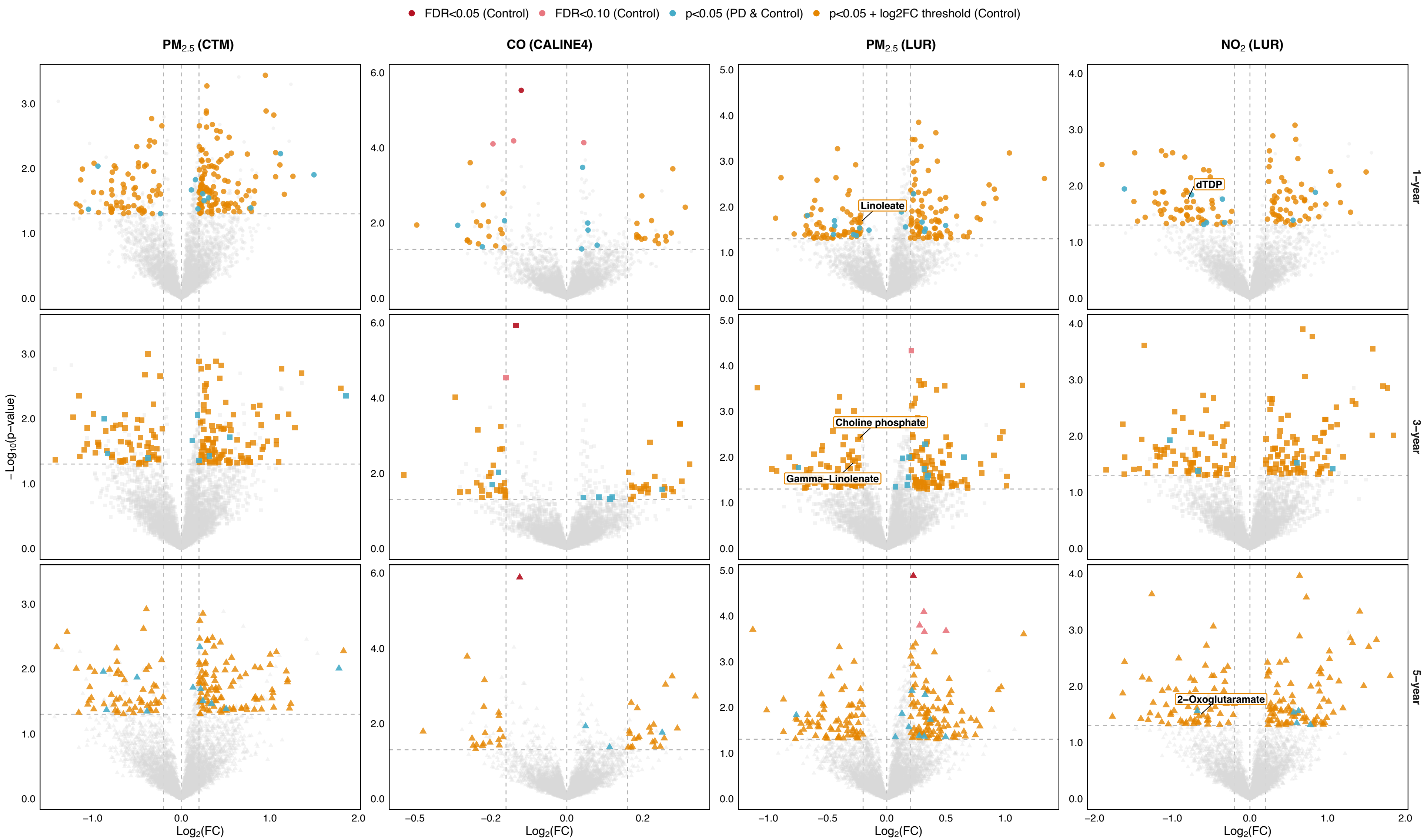

Supplementary Figure 4. Metabolomic features associated with air pollution exposure in controls. Volcano plots showing $\log_2$ fold change ($\log_2$FC, x-axis) versus $-\log_{10}$ p-value (y-axis) for each metabolite feature in metabolome-wide associations between serum metabolites and air pollution exposure in controls. Panels show four exposure models: a) $PM_{2.5}$ estimated by a chemical transport model (CTM); b) carbon monoxide (CO) by the California Line Source Dispersion Model, version 4 (CALINE4); c) $PM_{2.5}$ by land use regression (LUR); and d) nitrogen dioxide (NO_2) by LUR. Within each panel, the three rows correspond to 1-year, 3-year, and 5-year average exposures prior to serum collection. Point shapes indicate the exposure window (circles, 1-year; squares, 3-year; triangles, 5-year). Point colors indicate statistical significance: red, false discovery rate (FDR)<0.05 in controls; pink, FDR<0.10 in controls; yellow, nominal p<0.05 in controls with |log2FC|>0.2; blue, nominal p<0.05 in both controls and PD patients. Annotated metabolite features are labeled within each panel. PD, Parkinson's disease; $PM_{2.5}$, particulate matter ≤2.5 µm.

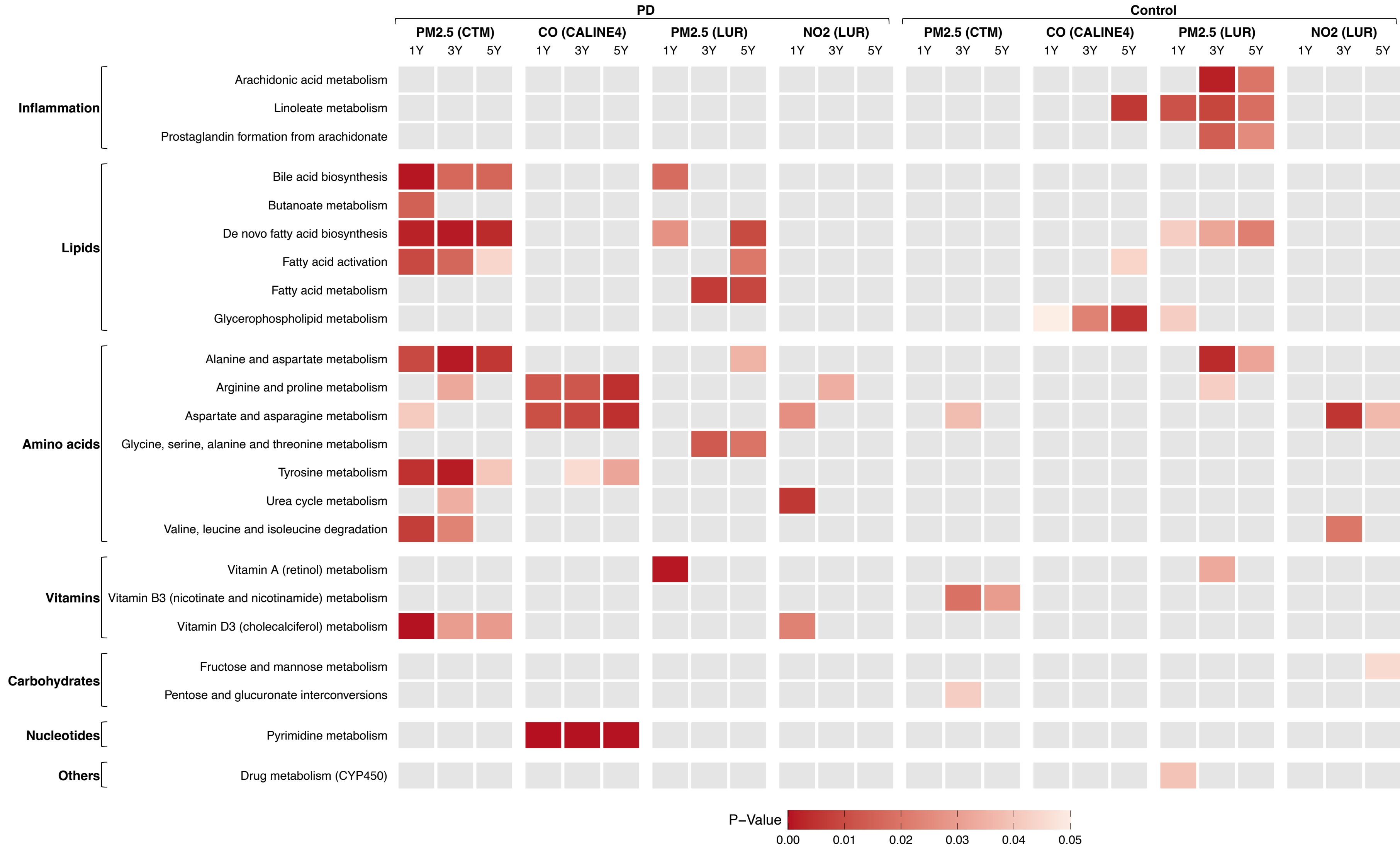

Supplementary Figure 5. Metabolic pathway enrichment by air pollution exposure across pollutants and exposure windows in PD patients and controls. Heatmap of metabolic pathway enrichment among metabolome-wide association study (MWAS) features associated with air pollution exposure (nominal $p<0.05$ with consistent direction across PEG1 and PEG2) in PD patients (left twelve columns) and controls (right twelve columns). Within each group, columns correspond to the four exposure models: $PM_{2.5}$ estimated by a chemical transport model (CTM), carbon monoxide (CO) by the California Line Source Dispersion Model, version 4 (CALINE4), $PM_{2.5}$ by land use regression (LUR), and nitrogen dioxide (NO_2) by LUR — each shown across 1-year (1Y), 3-year (3Y), and 5-year (5Y) average exposures prior to serum collection. Pathways were included if they contained ≥5 metabolites associated with air pollution and showed pathway overrepresentation at $p<0.05$. Pathways are grouped on the y-axis by functional class (inflammation, lipids, amino acids, vitamins, carbohydrates, nucleotides, and others). Cell color reflects the pathway enrichment p-value on a graded red scale, with darker red indicating lower p-values; grey cells indicate non-significant enrichment ($p\geq0.05$). PD, Parkinson's disease; PEG, Parkinson's Environment and Gene; $PM_{2.5}$, particulate matter ≤2.5μm.
